# Early detection of deep pelvic surgical site infection by microdialysis after abdominoperineal resection for locally advanced rectal cancer

**DOI:** 10.1007/s10151-025-03156-w

**Published:** 2025-05-29

**Authors:** J. Asvall, H. Haugaa, S. G. Larsen, T. F. R. Skarholt, B. M. Botnen, K. Flatmark, T. I. Tønnessen, E. B. Thorgersen

**Affiliations:** 1 Department of Research and Development, Division of Emergencies and Critical Care, Oslo University Hospital, Oslo, Norway; 2 Institute of Clinical Medicine, University of Oslo, Oslo, Norway; 3 Department of Anesthesia and Intensive Care Medicine, Oslo University Hospital, Oslo, Norway; 4 Lovisenberg Diaconal University College, Oslo, Norway; 5 Department of Surgical Oncology, Section of Abdominal Cancer Surgery, The Radium Hospital, Oslo University Hospital, Oslo, Norway; 6 Department of Anesthesia, Intensive Care Medicine and Operating Services, Oslo University Hospital, The Radium Hospital, Oslo, Norway; 7 Division of Emergencies and Critical Care, Department of Anesthesia, Intensive Care Medicine and Operating Services, Section of Anesthesia and Intensive Care Medicine, The Radium Hospital, Oslo University Hospital, Oslo, Norway

**Keywords:** Rectal cancer, Chemoradiotherapy, Abdominoperineal resection, Surgical site infection, Microdialysis, Lactate

## Abstract

**Background:**

Patients with locally advanced rectal cancer (LARC) treated with (chemo)-radiotherapy before abdominoperineal resection (APR) are at high risk of developing pelvic organ/space surgical site infection (O/S-SSI). This increases morbidity and prolongs length of stay. Vague symptoms delay diagnosis. In microdialysis, thin catheters are placed in tissue enabling monitoring of metabolism. We hypothesize that local metabolic changes related to O/S-SSI might be detected by microdialysis.

**Methods:**

In a prospective observational study, 38 patients who underwent open APR for LARC were analysed. At the end of surgery microdialysis catheters were placed in remnant tissue of the pelvic floor. Postoperatively, metabolic parameters including lactate, pyruvate, glucose and glycerol were measured, and the lactate-to-pyruvate (L/P) ratio was calculated. Out of 38 patients, 12 (32%) developed O/S-SSI.

**Results:**

O/S-SSI was diagnosed median 9 (range 6–17) days after surgery. On the day of surgery, mean lactate in the O/S-SSI group was 6.0 mmol/L, whereas it was 3.6 mmol/L in the no-O/S-SSI group. ROC analysis (AUC = 0.73), with cut-point lactate 5.7, detected O/S-SSI with 92% sensitivity and 65% specificity. Overall mean lactate was 1.9 mmol/L higher in the O/S-SSI group than in the no-O/S-SSI group (*P* = 0.002). Overall mean L/P ratio was 34 units higher in the O/S-SSI group (*P* = 0.001).

**Conclusions:**

In patients developing pelvic O/S-SSI, tissue lactate and L/P ratio measured by microdialysis were significantly higher and evident already from the day of surgery, 9 days prior to diagnosis, with high negative predictive value and moderate positive predictive value. Local monitoring using microdialysis may aid detection of O/S-SSI.

## Introduction

To improve local control, rectal cancers threatening the mesorectal fascia, invading adjacent organs and structures or with lateral metastatic lymph nodes (LLN) are given neoadjuvant therapy prior to surgery [1, 2]. Abdominoperineal resection (APR) is a comprehensive surgery in the pelvis, with a surgical wound both on the abdominal and the perineal side, leaving parts of the pelvis empty [3].

The incidence of deep pelvic organ/space surgical site infection (O/S-SSI) after APR is high, particularly after neoadjuvant (chemo-) radiotherapy ((C)RT) [4, 5]. O/S-SSI may lead to prolonged morbidity [6]. Out of 249 patients who underwent APR, 25% experienced O/S-SSI and 54% had incomplete perineal wound healing after 3 months [5]. Overall and cancer-specific survival rates might be reduced after O/S-SSI [6–8]. As vague symptoms delay diagnosis, better diagnostic tools may shorten the degree of this complication. Preoperative radiotherapy is shown to increase the frequency of O/S-SSI and reduce microcirculation [9, 10], thus ischemia seems to be a risk factor. Typical metabolic changes in ischemic tissue are elevated lactate and increased lactate-to-pyruvate (L/P) ratio, detectable with microdialysis and might predict infection [11]. By positioning microdialysis catheters in the area of interest, close to real-time monitoring of local metabolism is achieved [12–15]. To our knowledge an explicit comparison of microdialysis with standard diagnostic tools is not performed, but a review has summarized the technique in a colorectal anastomotic leakage setting, and concluded it may be useful [16]. In the present study, feasibility of detection of O/S-SSI by microdialysis after APR is explored.

We hypothesized that local tissue metabolism measured by microdialysis in patients evolving deep pelvic O/S-SSI differed compared with those without O/S-SSI.

## Methods

### Study design and population

The study was a single-centre, prospective observational study. A total of 39 patients older than 18 years of age with primary advanced rectal adenocarcinoma having received radiation ≥ 25 Gy to the pelvis, scheduled for open APR by the multidisciplinary team, were included between October 2016 and December 2021. One patient was excluded because of a perioperative perineal burn injury. Data from the remaining 38 patients were used in further analyses.

The project was approved by the Regional Ethics Committee of South-East Norway (2016/865) with amendment (document-id 1,191,974, approved 27.06.19). Oral and written informed consent was obtained before patient inclusion. The study is registered in the Clinical Trials registry (NCT03392584).

### Microdialysis

The microdialysis system consists of a syringe pump infusing a physiological solution through a catheter with an inlet and an outlet tube. At the distal part, a semipermeable membrane allows for diffusion of substances into the catheter and collection in a microvial. At the end of surgery, the right abdominal wall was punctured by a needle through which two microdialysis catheters were passed and inserted into the remaining muscular tissue of the pelvic floor guided by a Secalon® 14G needle from the perineal side. The position was superficially intramuscular, not freely intraperitoneally, to avoid migration from the pelvic cavity, and in the area where the O/S-SSI usually emerge. The catheter membranes had a 100 kDa pore size (CMA 65, M Dialysis AB, Stockholm, Sweden), and were perfused with 6% hydroxyethyl starch at a rate of 1 µL/minute by microinjection pumps (CMA 107, M Dialysis AB, Stockholm, Sweden) [17]. The samples were collected in 200 μL plastic microvials (M Dialysis AB, Stockholm, Sweden).

The microvials were analysed for lactate, pyruvate, glycerol and glucose every third hour between 09:00 and 24:00 using an on-site microdialysis analyser (Iscus Flex, M Dialysis AB, Stockholm, Sweden).

### Preoperative assessment and routine blood samples

Preoperative assessment comprised computed tomography (CT) of chest, abdomen and pelvis; pelvic magnetic resonance imaging (MRI); and proctoscopy. Blood to analyse C-reactive protein (CRP) and white blood cell count (WBC) was sampled prior to surgery and thereafter every 1–2 days depending on the clinical status of the patient.

### Definition and management of deep pelvic O/S-SSI

Deep pelvic O/S-SSI was defined as a collection of fluid in the pelvis on CT with concurrent clinical or biochemical signs of infection as described in detail previously [5]. Further, we used the grading system of ECDC [18]. CT was performed in the case of clinical suspicion of O/S-SSI. Routine treatment of O/S-SSI was antibiotics in combination with percutaneous drainage.

### Data presentation, data processing and statistical analyses

In the power calculation we assumed an incidence of O/S-SSI of 15%, a normal lactate value of 1.5 mmol/L versus a pathological value of 2.5 (95% CI 1.8–3.2) mmol/L, a power of 0.80, and a required *P*-value of < 0.05. On the basis of this calculation, a minimum of 28 patients was needed in the study to compare differences in lactate between patients evolving pelvic O/S-SSI and those who did not. Single data points in the microdialysis data obviously out of range were replaced by average values in the case of stable values before and after, otherwise set as missing. Single L/P ratios, > 500, as a result of very low pyruvate, were set as missing. In the case of two functioning catheters, average values of the two were calculated. Data were assumed to be non-normally distributed, thus Mann–Whitney *U* test was used in descriptive statistics to compare the groups. For the microdialysis data, a linear mixed model analysis for repeated measurements was chosen. The* P*-value represents differences for the observation period as a whole. To evaluate differences in metabolic markers on the day of surgery and to calculate the day CRP differed between the groups, a mixed model pairwise comparison was used. A *P*-value of < 0.05 was considered statistically significant. Post hoc receiver operating characteristics (ROC) analysis of maximum values of lactate and L/P ratio on the day of surgery was used to describe the discrimination accuracy of these parameters. Cut-points were chosen on the basis of distance to the upper left corner of the ROC-curve and clinical relevance. Statistical analyses were conducted using STATA software (version 18. StataCorp LCC, Texas, USA). Figures were designed using GraphPad Prism (version 10.1.2. GraphPad Software, Inc, California, USA).

## Results

### Patient characteristics

Apart from cardiovascular disease (0% in the O/S-SSI group versus 27% in the no-O/S-SSI group, *P* = 0.047), there were no significant differences between the groups (Table 1).

**Table 1:** Patient characteristics

	O/S-SSI^a^ (*n* = 12)	no-O/S-SSI (*n* = 26)	*P*-value^b^
Gender			0.40
Female	2 (17)^c^	2 (8)	
Male	10 (83)	24 (92)	
Age	59 (47–83)^d^	67 (26–79)	0.75
BMI^e^	28 (21–45)	25 (16–40)	0.08
ASA^f^			0.28
ASA 1–2	11 (92)	20 (77)	
ASA 3–4	1 (8)	6 (23)	
ECOG^g^			0.49
ECOG 0–1	12 (100)	25 (96)	
ECOG 2–4	0 (0)	1 (4)	
Smoker	1 (8)	6 (23)	0.28
Cardiovascular disease	0 (0)	7 (27)	0.047
Obstructive pulmonary disease	1 (8)	3 (12)	0.76
Diabetes	1 (8)	3 (12)	0.76
cTNM stadium^h^			
cT			0.094
T2	0 (0)	1 (4)	
T3	10 (83)	12 (46)	
T4b	2 (17)	13 (50)	
cN			0.084
N0	2 (17)	9 (35)	
N1	5 (42)	7 (27)	
N2	0 (0)	6 (23)	
LLN^i^	5 (42)	4 (15)	
cM			0.12
cM0	9 (75)	24 (92)	
cM1	3 (25)	1 (4)	
cMx	0 (0)	1 (4)	
ypTNM stadium			
ypT			0.55
T0	1 (8)	6 (23)	
T2	4 (33)	7 (27)	
T3	7 (58)	13 (50)	
ypN			0.045
N0	5 (42)	19 (73)	
N1	5 (42)	7 (27)	
LLN	2 (17)	0 (0)	
ypM			0.14
M0	9 (75)	24 (92)	
M1	3 (25)	2 (8)	
Radiotherapy			0.73
Short course 25 Gy	3 (25)	5 (20)	
Long course 50 Gy	9 (75)	20 (80)	
Time from diagnosis to surgery (days)	128 (95–301)	128 (11–288)	0.74
Time from radiotherapy to surgery (days)	63 (41–95)	62 (39–193)	0.89
CEA^j^ (prior to surgery)	5 (1–35)	2 (1–10)	0.2
CRP^k^ (prior to surgery)	2 (1–21)	1 (1–10)	0.17

^a^Organ/space-surgical site infection

^b^Pearson’s chi-squared, except age, body mass index (BMI), time from diagnosis to surgery, time from radiotherapy to surgery, CEA and CRP (Mann–Whitney *U* test). (The chi-squared tests in this table indicate whether there are significant differences in distribution of the categories of the tested parameter.)

^c^Number of patients and percentage

^d^Median and range

^e^Body mass index

^f^American Society of Anesthesiologists Physical Status Classification System

^g^Eastern Cooperative Oncology Group performance status

^h^TNM Classification of Malignant Tumours (TNM8). (cTNM – before radiotherapy, ypTNM – histology)

^i^Lateral lymph nodes

^j^Carcinoembryonic antigen – measured prior to surgery

^k^C-reactive protein – measured prior to surgery

### Staging and neoadjuvant treatment

There were no significant differences in MRI tumour staging before neoadjuvant treatment (cTNM-stadium) or distribution of neoadjuvant (C)RT, either 2 Gy × 25 or 5 Gy × 5, between the groups (Table 1).

### Operations and hospital length of stay

Neither operating time nor blood loss differed between the groups (Table 2). The median length of stay for the O/S-SSI group was longer, 18 days compared with 10 days for the no-O/S-SSI group (*P* = 0.001).

**Table 2:** Pre- and postoperative patient characteristics

	O/S-SSI^a^ (*n* = 12)	no-O/S-SSI (*n* = 26)	*P* value^b^
Operating time (min)	328 (188–432)^c^	253 (167–513)	0.15
Operative blood loss (mL)	500 (200–2300)	400 (150–2500)	0.28
Length of stay (days)	18 (9–31)	10 (7–28)	< 0.001
Complications other than O/S-SSI			
Midline wound infection (S-SSI)^d^	1 (8)^e^	3 (12)	
Perineal wound infection (S-SSI)	3 (25)	0 (0)	
Infection of unknown origin	0 (0)	2 (8)	
Urinary tract infection	1 (8)	2 (8)	
Respiratory failure	2 (17)	2 (8)	
Pulmonary embolism	1 (8)	1 (4)	
Redo surgery	1 (8)	2 (8)	
(No complications other than O/S-SSI)	5 (42)	9 (35)	
Accordion^f^—expanded classification			< 0.001
Accordion < 3 or no complications	1 (8)	21 (81)	
Accordion 3–4	9 (75)	3 (12)	
Accordion 5–6	2 (17)	2 (8)	
Perineal dehiscence after 3 months			0.16
No	5 (42)	18 (69)	
Yes	7 (58)	7 (27)	
Unknown	0 (0)	1 (4)	

^a^Organ/space-surgical site infection

^b^Pearson’s chi-squared, except operating time, operative blood loss and length of stay (Mann–Whitney *U* test) (The chi-squared tests in this table indicate whether there are significant differences in distribution of the categories of the tested parameter)

^c^Median and range

^d^Superficial incisional – surgical site infection

^e^Number of patients and percentage

^f^The Accordion Severity Grading System of Surgical Complications

### Routine blood samples

**Fig. 1:**
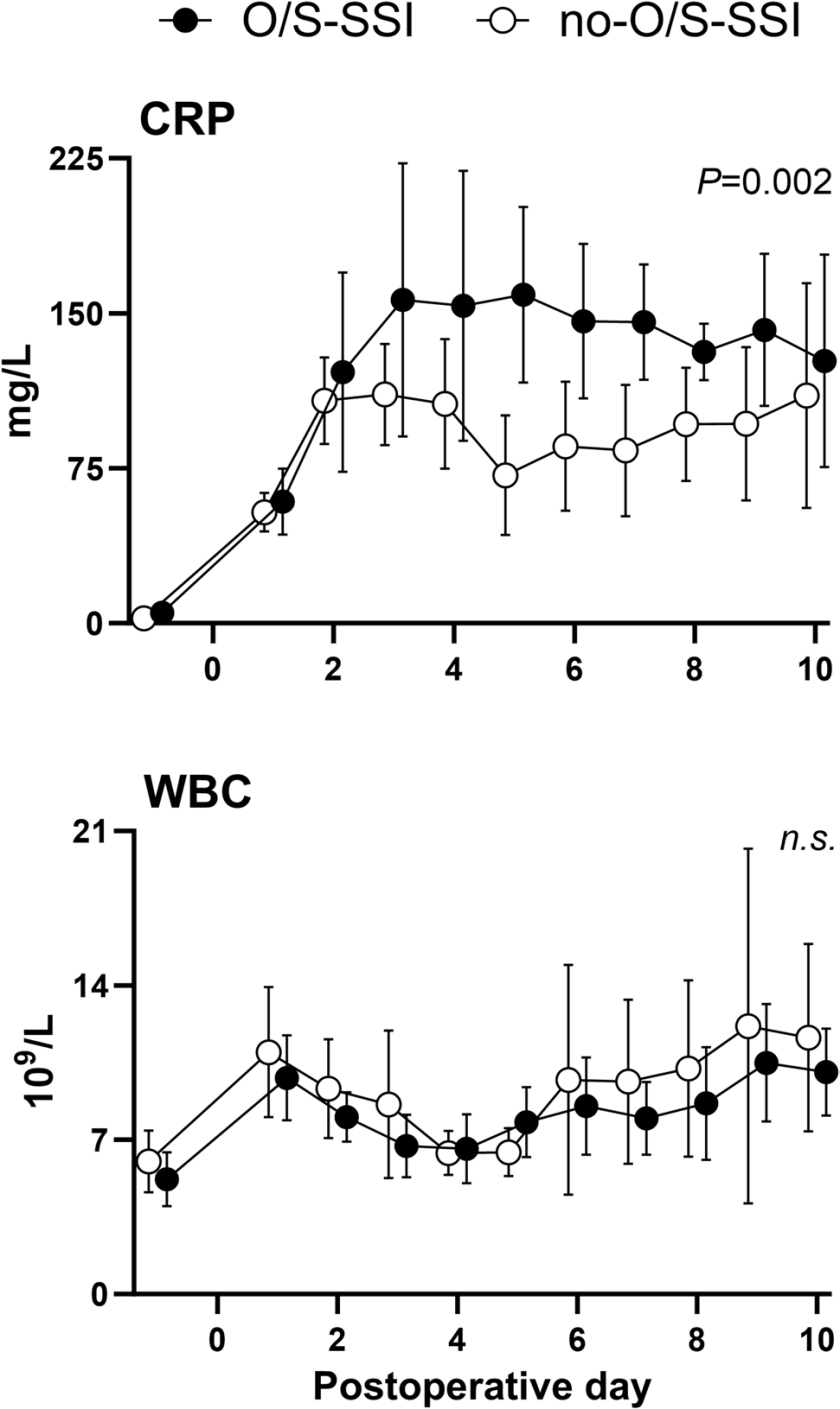
CRP and WBC in patients with and without deep pelvic organ/space surgical site infection (O/S-SSI) after APR. Daily measurements from the day before surgery until 10 days after surgery in *n* = 12 patients with deep pelvic O/S-SSI (O/S-SSI) and *n* = 26 patients without deep pelvic O/S-SSI (no-O/S-SSI). Data are presented as mean values with 95% confidence intervals per day. Black circles = O/S-SSI. White circles = no-O/S-SSI. *P*-values < 0.05 were considered statistically significant

Preoperative CRP levels were equal (Fig. 1). After surgery, CRP increased in both groups until the second postoperative day, followed by a modest rise in the O/S-SSI group. Overall CRP was 42 mg/L higher in the O/S-SSI group (*P* = 0.02) and there was a significant difference between the groups from the fourth postoperative day. WBC did not differ between the groups (Fig. 1).

### Pathology

There was no significant difference between the groups with respect to T-stage, but there was a shift towards higher N-staging among those with O/S-SSI (*P* = 0.045) (Table 1).

### Detection and management of pelvic O/S-SSI

In total, 12 out of 38 patients (32%) were diagnosed with pelvic O/S-SSI (O/S-SSI group) at median postoperative day 9 (range 6–17) (Table 3), and 11 of 12 patients in the O/S-SSI group were treated with radiologically assisted drainage from the day of diagnosis in addition to antibiotics. One patient was treated with antibiotics alone from postoperative day 9 due to clinical presentation, as the radiological examination was inconclusive. Another patient, discharged on postoperative day 8, was readmitted to the local hospital on postoperative day 22, with pelvic O/S-SSI being diagnosed and treated. This patient was not diagnosed with pelvic O/S-SSI during primary admission, hence their inclusion in the no-O/S-SSI group.

**Table 3:** Diagnosis, diagnostic signs and treatment of patients with pelvic organ/space surgical site infection (O/S-SSI)

Postoperative day (POD)														
Patient	Diagnosis	Clinical signs								Biochemical signs		CT	Treatment	
		Fever^a^	HR^b^	RR^c^	Reduced state^d^	Ileus^e^	Nausea	Vomiting	Abd pain^f^	CRP^g^	WBC^h^		Drain	Antibiotics
#1	6	1, 2	2	–	–	–	–	–	–	5	6	6	6	7
#2	9	0	–	–	3	3	3	3	–	6	9	3, 5	9	6
#3	7	2	–	3, 6	–	–	–	–	–	8	8	7	7	7
#4	9	6	–	–	–	15	7, 14	14	5, 8, 14	9	9	9	9	11
#5	17	–	5	–	15	–	–	–	2	6	6	17	17	15
#6	8	–	–	–	–	–	–	–	7, 11	7	4	8	8	9
#7	9	7	7	–	–	–	–	–	1	8	7	9	9	7
#8	9	–	7	2, 3, 7	5	–	8	–	8	7	6	8*	–	9
#9	6	2,6	2	–	2	–	3	–	3	6	6	2, 6	6	2
#10	16	–	–	5, 12	4	4	6	9	9	9	6	9, 16	16	9
#11	14	4	1	2, 8	9	–	–	–	7, 11	14	5	5, 14	14	14
#12	8	5	4	–	5	–	–	–	–	9	5	5	8	8

Overview of diagnosis of pelvic O/S-SSI (*n* = 12) and treatment. Diagnosis based on CT findings with concurrent clinical and biochemical signs of infection. Numbers indicate postoperative day. *Apart from patient #8, whose CT findings on POD8 were unclear, drainage was omitted and antibiotics were started on POD9, and the remaining 11 cases were treated with drain on the day of diagnosis. Antibiotics were started before diagnosis in 5 patients, on the day of diagnosis in 4 patients and after diagnosis in 3

^a^Fever: T > 38C

^b^Heart rate (HR) > 90

^c^Respiratory rate (RR) > 20

^d^Reduced general condition (e.g. exhaustion, fatigue, chills, confusion, discomfort)

^e^Ileus – clinical symptoms or radiological findings

^f^Abdominal pain

^g^Increased C-reactive protein (CRP) – day of increased value after first postoperative increase

^h^Increased white blood cell (WBC) count – day of increased value after first postoperative increase

### Complications other than pelvic O/S-SSI

Complications apart from deep pelvic O/S-SSI were midline and perineal superficial incisional SSI (S-SSI), urinary tract infection, infection of unknown origin, pleural fluid in need of drainage, pulmonary embolism and respiratory failure (Table 2). Complications were graded using the Accordion Severity Grading System of Surgical Complications [19]. Redo surgery was performed in one patient in the O/S-SSI group (ileus) and in two no-O/S-SSI patients (rupture of the abdominal fascia and necrotic stoma).

### Metabolism monitored by microdialysis

**Fig. 2:**
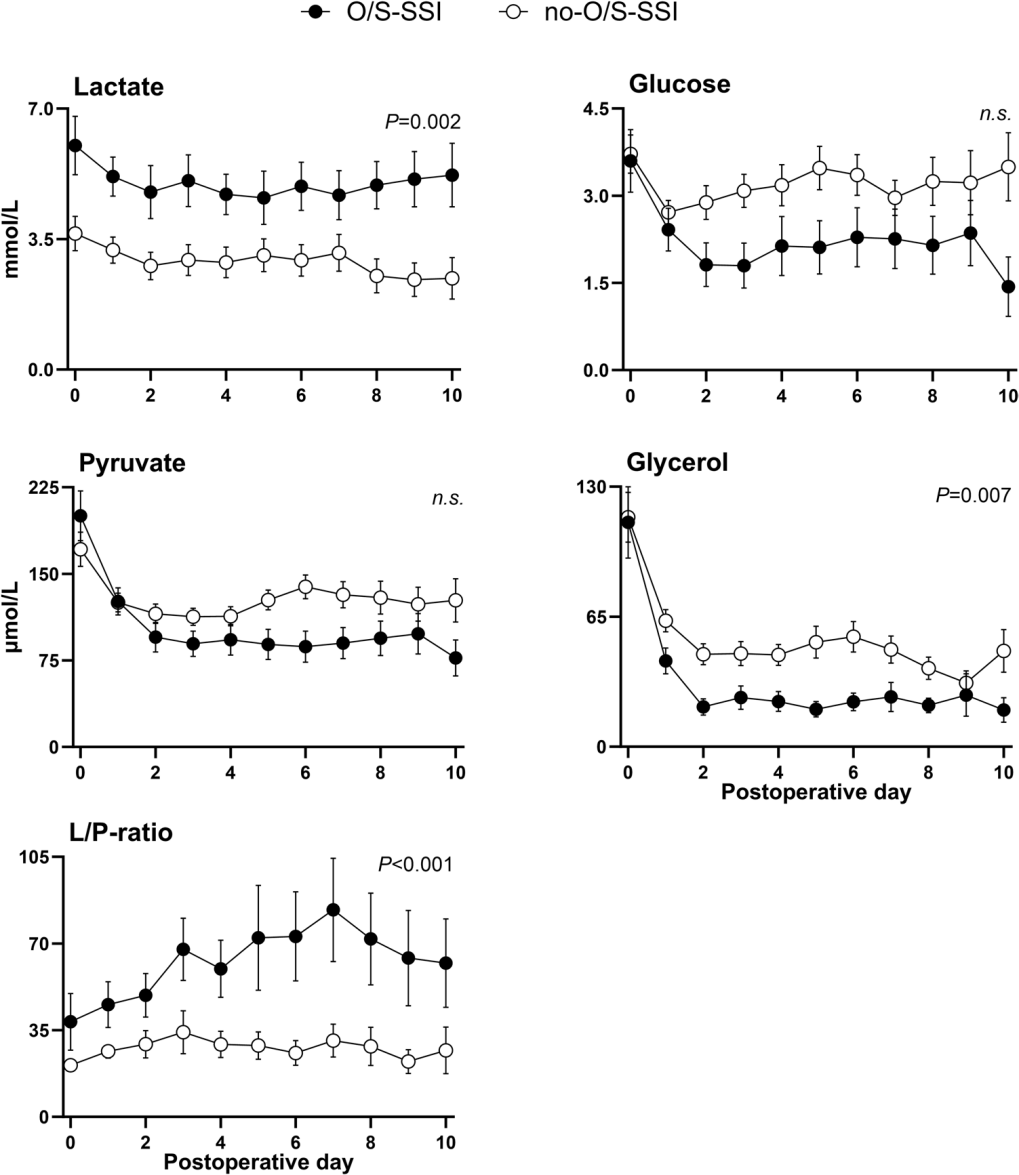
Lactate, pyruvate, L/P ratio, glucose and glycerol measured by microdialysis catheters in the pelvis in patients with and without deep pelvic organ/space surgical site infection (O/S-SSI) after APR. Microdialysis parameters were measured five times per day from the day of surgery to 10 days after surgery in *n* = 12 patients with deep pelvic O/S-SSI (O/S-SSI) and *n* = 26 patients without deep pelvic O/S-SSI (no-O/S-SSI). Data are presented as mean values with 95% confidence intervals of microdialysis measurements per day. Black circles = O/S-SSI. White circles = no-O/S-SSI. *P*-values < 0.05 were considered statistically significant. n.s., non-significant

**Fig. 3:**
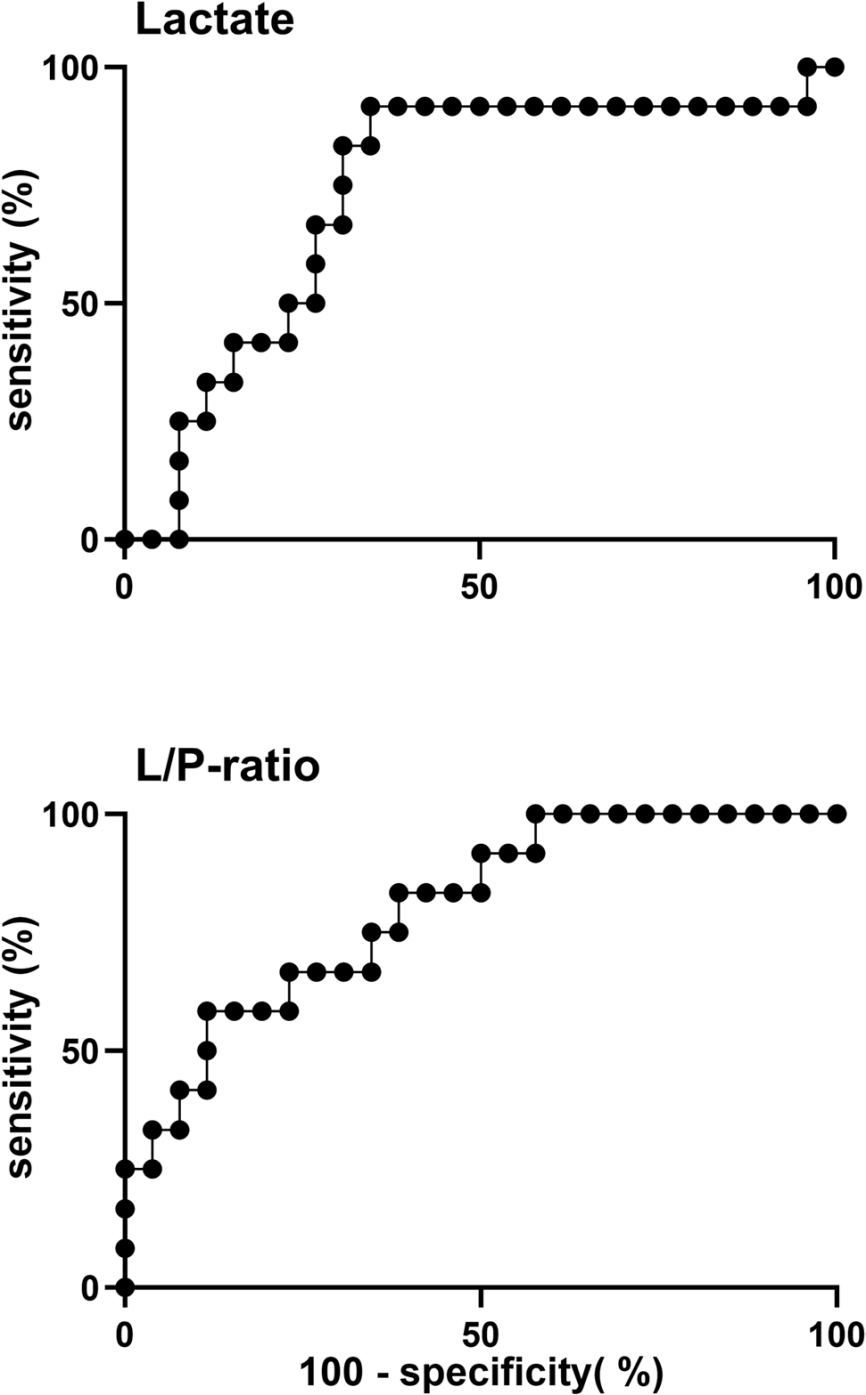
Receiver operating characteristics (ROC) analyses of lactate and L/P ratio measured by microdialysis catheters on the day of surgery. Lactate and L/P ratio’s abilities to detect deep pelvic organ/space surgical site infection (O/S-SSI) after APR. Maximum value through the day of surgery for each patient is compared with the diagnosis of deep pelvic O/S-SSI in *n* = 12 patients with *n* = 26 patients without deep pelvic O/S-SSI (no-O/S-SSI). Lactate AUC = 0.73 (95% CI 0.55–0.91). L/P AUC = 0.80 (95% CI 0.65–0.95).

On the day of surgery, lactate was 67% higher in the O/S-SSI group with a mean value of 6.0 (95% CI 5.2–6.8) mmol/L, whereas the mean value was 3.6 (95% CI 3.2–4.1) mmol/L in the no-O/S-SSI group (*P* < 0.001) (Fig. 2). This trend continued throughout the observation period of 10 days. The lactate values in both groups followed similar patterns, with a reduction from surgery until postoperative day 2, thereafter a stable period until postoperative day 7. Afterwards lactate increased in the O/S-SSI group, but decreased in the no-O/S-SSI group. The overall mean lactate value was 1.9 mmol/L higher in the O/S-SSI group (*P* = 0.002). ROC analysis of maximum lactate on the day of surgery (AUC = 0.73) with cut-point of lactate > 5.7 detected O/S-SSI with 92% sensitivity and 65% specificity, with corresponding positive predictive value (PPV) 66% and negative predictive value (NPV) 94% (Fig. 3).

Mean pyruvate on the day of surgery was higher in the O/S-SSI group, 200 (95% CI 179–222) µmol/L, versus 171 (95% CI 157–186) µmol/L in the no-O/S-SSI group (Fig. 2). Pyruvate values declined in both groups until postoperative day 4 and remained at the same level in the O/S-SSI group, whereas they increased slightly in the no-O/S-SSI group until postoperative day 10. From day 3, pyruvate levels were lower in the O/S-SSI group. The overall mean value was 17.3 µmol/L non-significantly lower in the O/S-SSI group.

Mean L/P ratio on the day of surgery was 81% higher in the O/S-SSI group, 38 (95% CI 27–50), versus 21 (95% CI 19–23) in the no-O/S-SSI group (*P* < 0.001) (Fig. 2).

L/P ratio increased in both groups from the day of surgery until postoperative day 3, thereafter declined in the no-O/S-SSI group, but continued to increase until postoperative day 7 in the O/S-SSI group. The average mean L/P ratio was significantly higher, with 34 units in the O/S-SSI group (*P* = 0.001).

ROC analysis of maximum L/P ratio on the day of surgery (AUC = 0.80), with cut-point of L/P ratio > 26, detected O/S-SSI with 92% sensitivity and 50% specificity, with corresponding PPV 46% and NPV 93% (Fig. 3).

The mean glucose levels were similar on the day of surgery, 3.6 (95% CI 3.1–4.1) mmol/L, versus 3.7 (95% CI 3.4–4.0) mmol/L in the no-O/S-SSI group (Fig. 2). Glucose declined in both groups between the day of surgery and postoperative day 1, but continued to decline in the O/S-SSI group until postoperative day 3. From postoperative day 3 to postoperative day 9, both groups followed the same pattern with a slight increase. Overall mean glucose was non-significantly lower with 0.84 mmol/L in the O/S-SSI group compared with the no-O/S-SSI group (*P* = 0.057).

The mean glycerol levels were similar on the day of surgery, 112 (95% CI, 94–130) µmol/L in the O/S-SSI group, versus 115 (95% CI 102–127) µmol/L in the no-O/S-SSI group (Fig. 2). Glycerol declined in both groups until postoperative day 9. The overall mean value was 21.4 µmol/L lower in the O/S-SSI group compared with the no-O/S-SSI group (*P* = 0.007).

Median duration of microdialysis catheters in situ was 8.3 days. In one no-O/S-SSI patient, the catheter was removed on postoperative day 3 during reoperation of the stoma and in one O/S-SSI patient, it was accidentally removed 4 days after surgery.

## Discussion

Lactate and L/P ratio in microdialysis fluid was significantly higher already from the day of surgery and persisted throughout the observation period in patients with deep pelvic O/S-SSI. On the basis of ROC analysis, performance of maximum lactate on the day of surgery to detect later development of O/S-SSI was moderate (AUC = 0.73), and good for L/P ratio (AUC = 0.80), but a cut-point of lactate > 5.7 had better PPV and NPV than L/P ratio > 26. As diagnosis by CT scan was performed 9 days after surgery, microdialysis may be an adjunct to detect pelvic O/S-SSI earlier than with standard diagnostics.

An immediate and thereafter continuous high level of lactate was measured in patients with O/S-SSI. On postoperative days 1–5, L/P ratio increased in the O/S-SSI group due to a decrease in pyruvate. Its sustained increase thereafter corresponded to a slight increase in lactate. These changes may partially derive from a metabolic shift from oxidative phosphorylation towards anaerobic glycolysis with increased lactate and reduced pyruvate among those with O/S-SSI. Anaerobic conditions may generate the increased lactate and reduced pyruvate, hence an increased L/P ratio. Such conditions may evolve with both reduced perfusion due to fibrotic impairment of local microcirculation after (C)RT or local increased metabolism caused by infection and inflammation [10, 20]. In our setting, both mechanisms are probably present. Combined increases in both lactate and L/P ratio are described after ischemia [21]. A study monitoring postoperative pancreatic fistulae after pancreatoduodenectomy with microdialysis observed early increases in lactate and L/P ratio and related this to inflammation and ischemia, similar to our findings [22]. Until quite recently anaerobic metabolism has been the proposed mechanism for changes in L/P ratio and lactate, but the relation between lactate and pyruvate seems more complex. It has been shown that stress hyperlactatemia also derives from increased aerobic lactate production, probably secondary to adrenergic stimulation [23]. Our measurements of initial high values of lactate and pyruvate in both groups may partly be related to the local stress response in tissue. Lactate also increases due to other mechanisms. Neutrophils prefer glycolytic metabolism when activated by infection. Thus aerobic glycolysis and fermentation of pyruvate to lactate instead of oxidative phosphorylation may contribute to reductions in glucose, decreased pyruvate, increased lactate and increased L/P ratio [24, 25]. As high lactate was observed already on the day of surgery, this may possibly primarily derive from ischemia and to a lesser extent from infection itself.

The increased lactate and L/P ratio associated with O/S-SSI in our study have similarities with previous microdialysis studies aimed to detect complications after various types of colorectal surgery other than APR. In a study of 23 patients undergoing anterior resection for rectal carcinoma, an increased intraperitoneal L/P ratio was detected on postoperative days 5 and 6 in seven patients with anastomotic leakage (AL) [26]. In these, the microdialysis catheters were placed close to the anastomosis and the changes were seen later than in our material, but before clinical symptoms. As we registered changes already from the day of surgery, this may reflect differences in the pathogenesis of AL versus pelvic O/S-SSI. Another study investigated AL after open and laparoscopic colorectal surgery and found isolated significant increased intraperitoneal lactate levels on the first 5 postoperative days [16]. In contrast to the former study [26], their observation of early increased lactate, which they related to ischemia, corresponds to our findings. Isolated significant increased intraperitoneal L/P ratio the first 48 postoperative hours in 16 of 60 patients developing complications, mainly AL, after major abdominal cancer surgery, is also described [27]. The differences in L/P ratio were modest, but present immediately after surgery, thus comparable to our findings. They suggested the increased L/P ratio was due to a change towards anaerobic metabolism and possibly the inflammatory response. Ellebæk et al. [28] monitored 45 patients with rectosigmoid cancer undergoing low anterior resection (LAR) for 5 days after surgery with microdialysis catheters placed intraperitoneally close to the anastomosis. In the four patients developing AL, overall L/P ratios were significantly increased, with a difference between the groups comparable to our study. In a follow-up prospective study in which 35 of 129 patients with rectal cancer developed AL after open or laparoscopic LAR [29], both maximum lactate and maximum L/P ratio intraperitoneally were significantly higher than in patients without AL. The patterns of changes in both lactate and L/P ratio were similar to our material. Taken together, it is probable that intra-abdominal monitoring of metabolic markers may reveal complications involving ischemia, inflammation and infection.

Impaired microcirculation after neoadjuvant (C)RT and APR resulting in an empty irradiated pelvis [30], in addition to the nature of the O/S-SSI, can contribute to our findings of significant and persisting increased levels of both lactate and L/P ratio. Intraperitoneal lactate has higher predictive value than a clinical score for AL [29], but O/S-SSI after APR has more subtle symptoms than AL. Clinical diagnosis of O/S-SSI may therefore be more challenging. As increased lactate and L/P ratio was measured early among patients later developing O/S-SSI, monitoring of these markers is promising as predictors of complication.

An overall borderline significant reduction in glucose in the O/S-SSI group was observed. Reduced glucose is earlier described in patients with AL after rectal surgery [28, 29]. An increased demand of glucose due to anaerobic conditions and inflammation-induced aggregation and activation of white blood cells probably contribute. Reduced glucose in tissue may raise suspicion of infection, but should not be used as a single marker.

There was a reduction of glycerol in the O/S-SSI group from the day after surgery. Glycerol is released through lipolysis or degeneration of phospholipids. In patients with AL after colorectal surgery, intraperitoneal glycerol levels were lower compared with patients with a patent anastomosis [31]. Another study found reduction in intraperitoneal glycerol the first 48 h in a group of patients with major abdominal surgery, before symptoms of complications evolved [27]. However, results are conflicting, as a study of AL after LAR showed no differences in glycerol [29]. In an animal experiment mimicking AL by intraperitoneal faecal instillation, glycerol levels were not affected throughout the observation period of 10 h [32], corresponding to our findings of equal values on the day of surgery. In our setting, a possible interpretation for the reduction from the first postoperative day is bacteria using glycerol as a source of energy [33, 34]. A finding of decreased glycerol should raise suspicion of infection.

CRP was overall significantly higher in the O/S-SSI group, with a significant difference between the groups from postoperative day 4, whereas no difference was registered in WBC count. In patients with pelvic O/S-SSI, we either observed a second increase in CRP after an initial postoperative increase, or that the initial increase did not decrease as expected. This is in line with previous research concluding that CRP is of limited value in detection of infection, at least until 3–5 days after surgery [35, 36]. A previous study combining immediate postoperative venous lactate with CRP 48 h after surgery demonstrated high NPV and moderate PPV in detection of O/S-SSI [37]. Adding lactate by microdialysis on postoperative day 1 to such a model may be of value in future studies.

Our study has several limitations. The small sample size increases the risk of type II errors, and it being a single-centre study affects external validity. Other complications than O/S-SSI, a wide range of operative times and bleeding, and possible dislocation of the microdialysis catheters, may have affected the microdialysis results. Initial intraperitoneal rather than intramuscular placement of the microdialysis catheters could therefore possibly have contributed to improved uniformity of the data. Hemodynamic instability and fluid resuscitation may be confounding factors. Analysing one patient, later evolving O/S-SSI in the no-O/S-SSI group to adhere to the protocol, may also bias the results. Individual differences in postoperative care as an Enhanced Recovery After Surgery (ERAS) protocol was not established at the time of the study and might have influenced the results as well.

To our knowledge, detection of deep pelvic O/S-SSI by microdialysis after neoadjuvant (C)RT followed by APR has not been studied. In patients with deep pelvic O/S-SSI, lactate and L/P ratio was higher from the day of surgery compared with patients without. ROC analyses revealed moderate-to-good performance for microdialysis to detect O/S-SSI with high NPV and moderate PPV. If confirmed by larger studies, the clinical implications of our findings should be increased focus on potential O/S-SSI in the case of high values, or supporting early discharge of patients in the case of low values. Our findings of early changes elucidate the benefits of local metabolic monitoring. Detecting and treating deep pelvic O/S-SSI early may prevent morbidity and reduce hospital length of stay.

## Data Availability

No datasets were generated or analysed during the current study.
